# Signatures Beyond Oncogenic Mutations in Cell-Free DNA Sequencing for Non-Invasive, Early Detection of Cancer

**DOI:** 10.3389/fgene.2021.759832

**Published:** 2021-10-14

**Authors:** Subhajyoti De

**Affiliations:** Rutgers Cancer Institute of New Jersey, New Brunswick, NJ, United States

**Keywords:** cancer, early detection, non-invasive, cell-free DNA, sequencing

## Abstract

Early detection of cancer saves lives, but an effective detection strategy in public health settings requires a delicate balance - periodic screening should neither miss rapidly progressing disease nor fail to detect rare tumors at unusual locations; on the other hand, even a modest false positive rate carries risks of over-diagnosis and over-treatment of relatively indolent non-malignant disease. Genomic profiling of cell-free DNA from liquid biopsy using massively parallel sequencing is emerging as an attractive, non-invasive screening platform for sensitive detection of multiple types of cancer in a single assay. Genomic data from cell-free DNA can not only identify oncogenic mutation status, but also additional molecular signatures related to potential tissue of origin, the extent of clonal growth, and malignant disease states. Utilization of the full potential of the molecular signatures from cfDNA sequencing data can guide clinical management strategies for targeted follow-ups using imaging or molecular marker-based diagnostic platforms and treatment options.

## Introduction

Early cancer diagnosis significantly reduces cancer mortality and improves the chances of a favorable response to treatment (Campbell et al., 2016). Currently, the routine early, screening for early detection is cancer type-specific and typically involves low dose computed tomography (CT), X-ray, or testing for cancer-specific molecular markers (e.g. tumor-specific antigen); some of these platforms have moderate accuracy and limited predictive power. For instance, In the National Lung Screening Trial (NLST) involving 53,454 persons at high risk for lung cancer at 33 U.S. medical centers, screening with low-dose computed tomography (CT) had a 96.4% false-positive rate. Unfortunately, a high false-positive rate translates to increased risks of over-diagnosis and over-treatment of relatively indolent non-malignant disease and reduces public confidence. Furthermore, a majority of these screening platforms are cancer-type specific, such that a negative screening result for a given cancer type does not eliminate the risk of undetected early malignancy elsewhere in the body. Therefore, an accurate, ideally non-invasive, pan-cancer screening strategy has been a long-standing interest in the clinical cancer research community, and can fundamentally benefit clinical management strategies for early detection of cancer.

Tumors release circulating tumor cells, cell-free DNA, and other cellular contents in blood and tissue-relevant body fluids (e.g. saliva, urine, and sputum for oral, bladder, and lung cancer respectively), and recent advances in genomics and multi-omics approaches have enabled sensitive detection of tumor-derived cell-free DNA with high specificity and sensitivity. As a major connective tissue, blood carries cell-free DNA and other molecules throughout the body, such that tumor-derived cell-free DNA for a majority of common cancer types can be detected in blood from a liquid biopsy. Indeed, next-generation sequencing-based molecular profiling of cell-free DNA (cfDNA) in blood has emerged as a promising, non-invasive method for advanced, high precision assessment of multi-cancer risk in a single assay (Crowley et al., 2013; Wan et al., 2017). Notably, in some cases, cfDNA profiling has detected cancer recurrence several weeks, months, or even years in advance relative to the current standard of care (Abbosh et al., 2017; Chen et al., 2020), which has far-reaching implications for early detection and clinical management.

**FIGURE 1:**
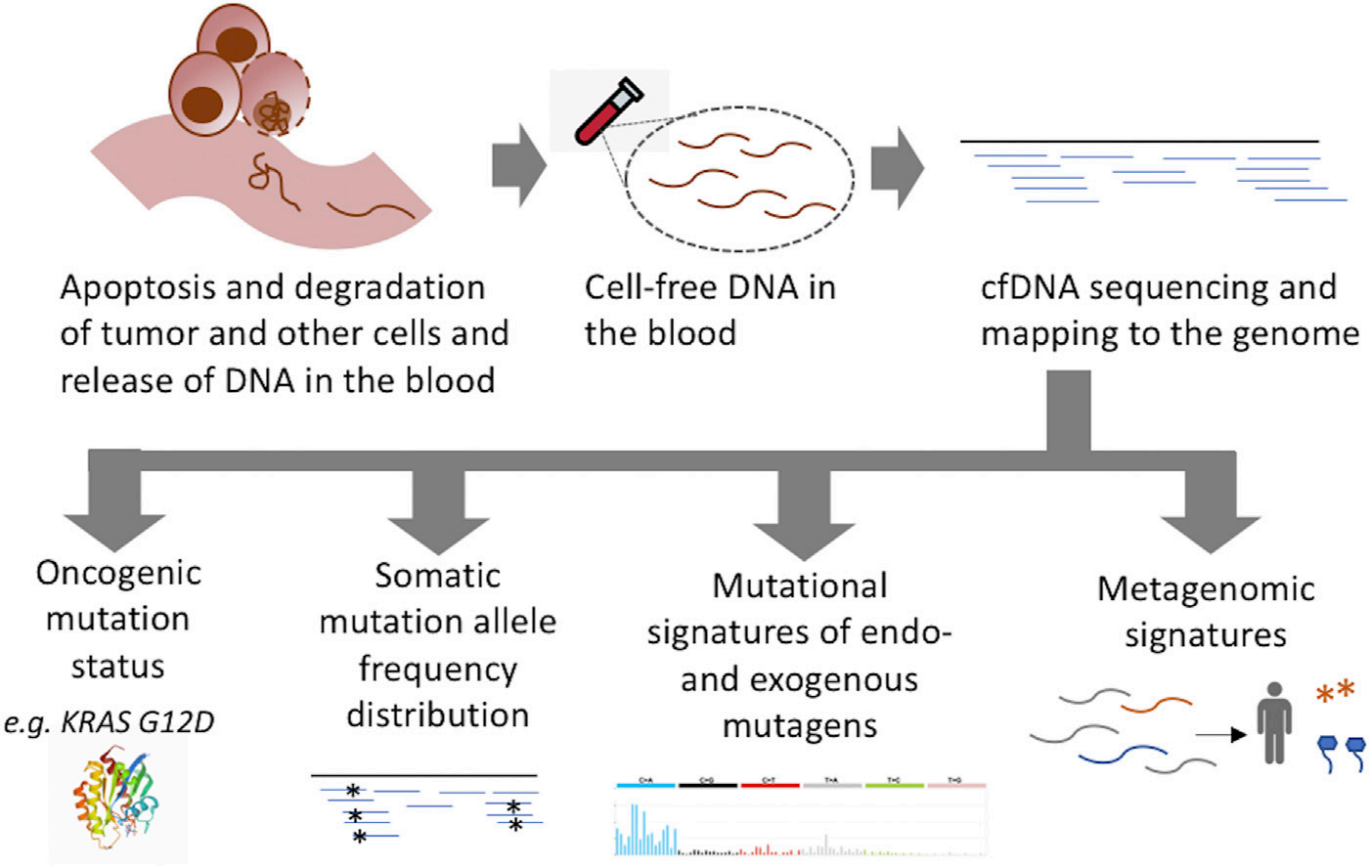
Molecular signatures in liquid biopsy sequencing relevant for early detection of cancer. Sequencing of cell free DNA can provide information about the status of cancer gene mutations as well as prevalent mutational signatures in somatic mutations in clonally amplified cell populations and microbial DNA, which can predict potential tissue of origin, the extent of clonal growth, and malignant disease status.

cfDNA profiling for tracking cancer progression in clinical genomic settings typically focuses on the detection of known oncogenic mutations using high depth sequencing of targeted cancer gene panel, and multiple FDA-approved tests are now available in the clinic (Ignatiadis et al., 2021). When the mutational makeup of the tumor is already known, it is cost-effective and ideal for tracking the progression of disease during treatment and potential relapse. However, when the genomic makeup of the tumor is unknown, as in the case of early detection, potential challenges need to be taken into consideration. First, oncogenic mutations are not unique to tumors; sensitive assays have routinely detected many cancer gene mutations in non-malignant tissues, especially in older individuals (Aghili et al., 2014; Alborelli et al., 2019; Liggett et al., 2019). Second, sequential accumulation of oncogenic mutations is a continuous process during pre-malignant and malignant transformation, such that oncogenic mutation status may not always identify the tipping point in cancer progression and/or the initiation of a malignant disease (De and Ganesan, 2017; Srivastava et al., 2018). Third, many oncogenic mutations are not cancer type-specific (Lawrence et al., 2013), and hence oncogenic mutation status alone may not uniquely identify the underlying cancer type. However, Genomic data from cell-free DNA also carries molecular signatures related to the cell of origin, malignant disease states, and the extent of neoplastic growth (Figure 1), which can complement information regarding oncogenic mutations for targeted follow-ups.

### Molecular Signatures in Cell-free DNA

Tumor and non-tumor tissues release cellular material including DNA fragments in the blood. cfDNA carries several attributes that are informative about the source tissue contexts, such that systematic analysis of those properties can provide further information about the disease potential and tissue of origin. First, a tumor genome has 10^3^−10^5^ somatic mutations, only a small minority of which are classic oncogenic mutations, while the others are passenger mutations − which are typically classified as the variants of unknown significance (VUS). Since cancer is a disease marked by clonal growth, the somatic mutations of malignant origin are likely to be present in clonally amplified cell populations (Podlaha et al., 2012); therefore unlike the baseline somatic mutations from other, non-tumor tissues, the cancer-associated mutations are likely to be present at a relatively higher allele frequency in cfDNA. Somatic mosaicism (Moss et al., 2018) i.e., presence of clonal growth in healthy somatic tissues, low tumor DNA fraction in cfDNA, as well as the low depth of coverage of cfDNA sequencing could potentially complicate the inferences. Nonetheless, the VUS vastly outnumber the oncogenic mutations, such that a joint analysis of allele frequency distributions of the VUS and oncogenic driver mutations from cfDNA sequencing data can provide increased statistical power to predict the presence of clonally amplified cell populations carrying the observed set of oncogenic mutations in the patient’s blood.

Second, the somatic mutations typically carry mutational signatures of tissue-dependent carcinogenic exposure and DNA repair defects. For instance, DNA derived from lung tissues e.g. in the liquid biopsy fraction typically carries mutational signatures of smoking and other pollutants (Abbosh et al., 2017). Since a liquid biopsy sample carries an admixture of DNA fragments derived from potential tumor tissues as well as hematopoietic and other non-malignant tissues (Hu et al., 2020), which are present even in healthy individuals, an analysis of the mutational signatures in cfDNA can help identify the presence of nucleic acid contents from unexpected tissue types (Gerstung et al., 2020). In addition, cfDNA fragments in the blood typically are 120−220 bp, or multiples thereof, with a maximum peak at 167 bp − which corresponds to the length of DNA wrapped around a single nucleosome in the source tissue, and therefore the genome-wide landscape of cfDNA fragments provides information about epigenome of the cell of origin.

Third, although cancer progression is a gradual process, often progressing through localized dysplasia and hyperplasia stages for years before neoplastic transformation, not all clonal growths progress to malignant disease (Podlaha et al., 2012; Gerstung et al., 2020; Singh et al., 2020). The neoplastic transformation typically involves overcoming replicative senescence and genomic instability, accompanied by invasive clonal growth. Genomic instability leads to distinct mutational signatures in somatic mutations, which are uncommon in normal tissues. For instance, the mutational signature SBS3, SBS8, and SBS40, which are associated with replication stress and genomic instability (Maley et al., 2017; Alexandrov et al., 2020; Gerstung et al., 2020) − rarely occur in normal or benign tissues, but are widely present in cancer genomes. Collectively, the presence of oncogenic mutations, as well as the somatic mutations with distinct mutational signatures and inferred chromatin makeups in cfDNA can help identify potentially malignant clonal growth in a narrow set of probable tissue types with significant contributions in the cfDNA.

Fourth, it is increasingly becoming evident that the tumor microenvironment does not only include tumor, immune, and stromal cells, but also diverse microbial entities including bacteria, fungi, and viruses (Poore et al., 2020). While earlier works identified tumor-associated microbiome predominantly in selected tumor types, recent studies suggest that tumor-associated microbiome might be present in most cancer types (Sr et al., 2020). Furthermore, microbial DNA from the tumor microenvironment could be detected alongside DNA from host tissues during unbiased total cfDNA sequencing from a liquid biopsy. Since different cancer tissue types have distinct microbial makeups (Sr et al., 2020), annotation of microbial DNA could provide additional clues about the potential sources of genetic material in blood. Indeed, it seems possible to correctly predict tumor types from microbial signatures in blood alone (Sr et al., 2020). Therefore, total DNA sequencing from blood can provide orthogonal metagenomic signatures characteristics of different cancer types, adding yet another line of evidence for the detection of the disease.

While the above attributes could be derived from the standard cfDNA sequencing, additional insights could be gained from profiling microRNA and DNA methylation states, and other biomarkers, which typically require dedicated assays. Taken together, in addition to the detection of oncogenic mutations the emerging molecular signatures in cfDNA sequencing (Figure 1) can provide multi-faceted insights into tumor etiology and disease status, and guide imaging or molecular marker-based targeted follow-ups and treatment options.

### Advances in cfDNA Sequencing and Genomic Analysis

A number of genomic approaches and computational methods have been developed for sensitive detection of somatic mutations and other molecular signatures from cfDNA sequencing. Recent developments have increased the yield and quality of cfDNA through a rational design (De, 2011), and it has been possible to isolate tumor-derived DNA from diverse types of body fluids including sputum, saliva, and urine to inform about cancer progression (Ignatiadis et al., 2021; Jj et al., 2020). In parallel, using specialized genomic and computational approaches it is now possible to detect somatic mutations at an ultra-low allele frequency (Mw et al., 2016; Sr et al., 2020). Moreover, integrative analysis of driver and passenger somatic mutations from cfDNA has been able to infer clonal and subclonal mutations, and also clonal architecture in the primary tumor for some patients (Abbosh et al., 2017)–which opens up the possibility of tracking clonal dynamics in vivo in real-time. Elegant computational analyses of cfDNA fragment patterns in patient samples have provided quantitative estimates of the likely tissue of origin of cfDNA (Schwarzenbach et al., 2011). More recently, Poore et al. used an innovative approach to identify cancer types based on microbial signatures in blood (Cc et al., 2020), which has significance for cancer diagnostics. Collectively, these and other advances provide a rich toolbox to track cancer progression non-invasively from blood, even at an early stage of the disease and recurrence.

## Discussion

Genomic profiling of cfDNA for diagnostic purposes, especially at an early stage of cancer is not trivial. Tumor DNA fraction in cfDNA in blood might be very low during the early non-invasive stage of the disease and can vary considerably, depending on the location of the tumor in the tissue contexts. It also remains challenging to assign the cell of origin to individual cfDNA molecules, potentially complicating etiological inferences. Large cohorts are required to assess the cancer type-specific accuracy of cfDNA profiling in the early detection setting. In any case, the benefits appear to outweigh the potential limitations. The technologies are maturing rapidly, and non-invasive cancer progression using cfDNA profiling has already moved from bench to bedside. A number of FDA-approved providers routinely offer services, such as the Guardant360 and FoundationOne Liquid CDx platforms to track cancer progression using liquid biopsy sequencing. This year, GRAIL Inc. has launched the Galleri platform under a CLIA-waiver and can be used to screen people of age 50 years or older at elevated risk of cancer.

While the current focus remains on the detection of somatic mutations in liquid biopsy, the signatures discussed above could be extracted from the clinical genomics workflow for cfDNA sequencing without additional sample preparation and processing and can provide additional insights that could further guide clinical decision-making. While liquid biopsy-based tracking of cancer progression is already integrated into the clinical management for cancer patients, leveraging it for non-invasive screening for early detection of pan-cancer risk at a low cost in a standardized setting will have important implications for clinical management of high-risk patient population and public health at large.

## Data Availability

The original contributions presented in the study are included in the article/Supplementary Material, further inquiries can be directed to the corresponding author.
